# Associations between Active Commuting to School and Health-Related Physical Fitness in Spanish School-Aged Children: A Cross-Sectional Study

**DOI:** 10.3390/ijerph120910362

**Published:** 2015-08-26

**Authors:** Emilio Villa-González, Jonatan R. Ruiz, Palma Chillón

**Affiliations:** 1 Department of Physical Culture, School of Health Sciences, National University of Chimborazo, Avda. Antonio José de Sucre, Km. 1 1/2 vía a Guano, 060150 Riobamba, Ecuador; 2 Profith “PROmoting FITness and Health through physical activity” Research Group, Department of Physical Education and Sport, School of Sport Science, University of Granada, Spain Ctra. Alfacar, s/n, 18011 Granada, Spain; E-Mails: ruizj@ugr.es (J.R.R.); pchillon@ugr.es (P.C.)

**Keywords:** physical activity, active transportation, public health, strength

## Abstract

Active commuting (walking or cycling) to school has been positively associated with improved fitness among adolescents. However, current evidence lacks information on whether this association persists in children. The aim of this study was to examine the association of active commuting to school with different fitness parameters in Spanish school-aged children. A total of 494 children (229 girls) from five primary schools in Granada and Jaén (Spain), aged between eight and 11 years, participated in this cross-sectional study. Participants completed the Assessing Levels of Physical Activity (ALPHA) fitness test battery and answered a self-reported questionnaire regarding the weekly travel mode to school. Active commuting to school was significantly associated with higher levels of speed-agility in boys (*p* *=* 0.048) and muscle strength of the lower body muscular fitness in girls (*p* *=* 0.016). However, there were no significant associations between active commuting to school and cardiorespiratory fitness and upper body muscular fitness. Our findings suggest that active commuting to school was associated with higher levels of both speed-agility and lower body muscular fitness in boys and girls, respectively. Future studies should confirm whether increasing active commuting to school increases speed-agility and muscle strength of the lower body.

## 1. Background

Physical fitness, mainly cardiorespiratory fitness, muscular fitness, and motor fitness, has shown to be a powerful marker of health in young people [1]. Low fitness has been associated with clustering of metabolic risk factors in young people that may persist into adulthood [2,3]. Modifying risk factor levels in children may thus be of critical importance for ameliorating future cardiovascular disease (CVD) risk [4,5]. 

Observational studies have shown that young people who actively commute to school (defined as the use of active means, such as walking and bicycling, to and from school) tend to be more physically active than passive commuters [6,7], although the contribution of active commuting to youths’ physical fitness levels (beyond aerobic fitness) is still unclear [8,9]. The association between active commuting to school and health-related physical fitness has been studied in adolescents [10,11,12] as well as in children [7,13,14,15]. These studies differed in the fitness components measured. All the previous studies measured cardiorespiratory fitness and a few of them measured muscular fitness [13,15] and speed-agility [13]. Active commuting by cycling has been associated with a higher cardiorespiratory fitness level compared to non-cyclist and walker adolescents [7,10,13]. Longitudinal studies have also shown that aerobic fitness improved from childhood to adolescence when participants switched from passive commuters to cycling as the dominant mode of travel to school [10,14].

Most of the studies that reported associations between active commuting to school and health-related physical fitness were targeted at adolescents and they focused mainly on cardiorespiratory fitness outcomes. However, few studies to date have explored the relationship between active commuting and health-related physical fitness in school-aged children (8–11 year olds), although there is previous evidence that recommends disease prevention should begin from childhood [16]. Thus, the current study aims to examine the association between active commuting to school and health-related physical fitness as measured by the Assessing Levels of Physical Activity (ALPHA) fitness test battery [17] in Spanish school-age children. Therefore, our hypothesis is that active commuters could show greater health-related physical fitness than passive commuters.

## 2. Methods

### 2.1. Participants and Study Design

A total of 494 children (229 girls) aged between 8 and 11 years (mean = 9.2 and SD = 0.6) participated in the study. Participants were recruited from five schools in the provinces of Granada (Salobreña, N = 125; Huétor Vega, N = 95; Santa Fe, N =96; the city of Granada, N = 128) and Jaén (Castillo de Locubin, N = 50). Those participants who had valid data on commuting to school were included (N = 469).

The study was conducted within a public health initiative lead by Diputación de Granada (Área de Medio Ambiente). The purpose of this program was to promote safe and healthy ways of commuting from home to school. An agreement was signed by the school board, Diputación de Granada, and the municipalities. The school board, parents, and students were informed about the study and they agreed to participate. Written consent from parents was obtained. The measurements were taken at each of the schools during school hours in the month of January, in the academic year 2011/2012. The Medical Ethics Committee of Hospital Virgen de las Nieves (Granada, Spain) approved the study design, study protocols, and informed consent procedure (case number 817).

### 2.2. Mode of Commuting to School

Participants completed a self-report questionnaire regarding the latest weekly patterns of commuting to and from school (Monday to Friday). The modes of commuting were: walk, bike, car, motorcycle, and bus. Walking and biking were categorized as active commuting, whereas traveling by car, motorcycle, and bus was categorized as passive commuting. This questionnaire has been proposed as the most appropriate measurement for asking about mode of commuting to school after reviewing 158 studies within the scientific literature [18]. Children completed the questionnaire with the help of the teacher and the research team. The weekly frequency of commuting to school was expressed as numbers of active travels per week to and from school (range: 0 to 10). One variable of three categories (0–2 active travels *vs.* 3–7 active travels *vs.* 8–10 active travels) was created. 

### 2.3. Health-Related Physical Fitness

Physical fitness, anthropometric variables, and sexual maturation status were assessed by the ALPHA fitness test battery of high priority, previously described in detail and validated in children and adolescents [17]. All participants completed the test during their physical education class. The same researchers performed all the measurements. Measurements were organized in a circuit, and participants performed each test consecutively, except for the cardiorespiratory fitness test, where several participants performed it at the same time. Completion of the physical measurements took one hour per school group. Several physical fitness tests were performed.

Cardiovascular fitness was assessed by means of the 20-m shuttle run test, as described in detail by Leger *et al.* [19]. In brief, the participants were required to run between two lines 20 meters (m) apart while keeping pace with audio signals emitted from a prerecorded CD. The initial speed was 8.5 km/h, and the speed was increased by 0.5 km/h per minute. The test was completed when the participants failed to reach the end lines concurrent with the audio signals on two consecutive occasions, or when the participants stopped because of fatigue. The equations of Leger *et al.* [19], previously validated in children and adolescents, were used to estimate the maximum oxygen consumption (VO_2max_) from the test scores. 

Lower body muscular fitness was assessed by means of the standing long jump. The participant stood behind the starting line, with feet together, and pushed off vigorously and jumped forward as far as possible. The distance was measured from the take-off line to the point where the back of the heel nearest to the take-off line lands on the mat or non-slippery floor. The test was repeated twice, and the best score was retained (in cm). 

Upper body muscle strength was assessed by means of handgrip strength using a hand dynamometer with adjustable grip (TKK 5401 Grip D; Takey, Tokyo, Japan). Children were given a brief demonstration and verbal instruction for the test and, if necessary, the dynamometer was adjusted according to the child’s hand size as recommended [20]. The test was done in the standing position with the wrist in the neutral position and the elbow extended; children were given verbal encouragement to “squeeze as hard as possible” and apply maximal effort for at least two seconds (s). Two attempts per hand were performed, and the best score was used. The average of the best scores achieved by each hand was used in the analysis. 

Speed-agility was measured by the 4 × 10 shuttle run test. Two lines, at a distance of 10 m, and two cones drawn were placed at the distant line. The participants ran as fast as possible from the starting line, picked up one sponge at the distant line, returned to the starting line, and placed the cone on this line before repeating the same run and retrieving the second sponge. Two attempts were performed, and the best score was retained (in seconds). The agility measure was the time to complete this 40-m run with correct placement of the cones. 

### 2.4. Covariates

The distance from home to school was estimated using the Internet program Google Maps V.6, similar to the previous study [21]. The shortest network path between each student's home address and the school measured in meters was used.

The anthropometric variables measured were weight and height. Body mass index (BMI) was calculated as weight (in kilograms)/height (in m^2^) by standard procedures. Waist circumference (WC) was measured in cm, using an inelastic tape. Sexual maturation status was assessed according to the method described by the scientific literature [22] in a brief examination by a trained researcher. Privacy was maintained at all times. 

### 2.5. Statistical Analyses 

Differences in socio-demographic characteristics (sex, age, and distance) between participants who provided fitness data and those who did not were tested using chi-square for categorical variables and Mann-Whitney U test for non-normal continuous variables. The normality of the fitness test variables was studied using the Kolmogorov-Smirnov test. Since they were not normal, a log transformation was conducted for each fitness test. Linear regression analysis with robust standard errors was used to investigate the associations of active commuting to school (number of active travels per week) with fitness tests (20-m shuttle run, standing long jump, handgrip strength, and 4 × 10 shuttle run) adjusting for age, BMI, and distance from home to school. 

We categorized the active commuting variable (number of active travels) in three categories (0–2 active travels *vs.* 3–7 active travels *vs.* 8–10 active travels). These cutoff points provided homogeneous sample sizes in every category. In order to know the impact of the number of active travels in the fitness tests, differences between active commuting to school (categorical variable with three categories) and fitness tests were examined by one-way analysis of covariance (ANCOVA) adjusting for age, BMI, and distance. *Post hoc* Bonferroni tests were conducted when the variable of the three categories was included. All the analyses were performed separately for boys and girls. Analyses were performed using the Statistical Package for the Social Sciences (SPSS) (v. 18.0 for Windows, Chicago, IL, USA), and the level of significance was set to 0.05.

## 3. Results

Drop-out analyses did not find differences between those who provided fitness data and those who did not. Characteristics of the study sample by sex are shown in Table 1. The average number of active travels (mainly walking) per week was 4.4, and there were no sex differences.

**Table 1:** Descriptive characteristics of the study sample stratified by sex.

Variables	N	All Mean (SD)		N	Boys Mean (SD)		N	Girls Mean (SD)	
Age (years)	469	9.2	(0.6)	251	9.3	(0.6)	218	9.2	(0.6)
Weight (kg)	424	37.3	(10.1)	235	37.7	(10.3)	187	37.0	(9.8)
Height (cm) BMI (kg/m2)	410 410	140.5 18.6	(8.8) (3.7)	226 226	140.9 18.6	(8.7) (3.7)	182 184	140.2 18.5	(8.8) (3.7)
WC (cm)	423	67.2	(10.0)	235	67.7	(10.5)	188	66.7	(9.2)
Sexual maturation status (%) Stage 1 Stage 2 Stage 3 Stage 4	420	26.7 60.5 12.1 0.7		233	4.3 84.5 10.3 0.9		187	54.5 30.5 14.4 0.5	
Cardiorespiratory fitness VO_2max_ (mL/kg per min)	413	41.5	(4.3)	229	42.6	(4.6)	184	40.2	(3.6)
20-m shuttle run (stage)	413	1.9	(1.3)	229	2.3	(1.5)	184	1.5	(1.0)
Muscular fitness Standing long jump (cm) Handgrip strength (kg)	420 423	114.1 14.4	(22.4) (6.7)	233 234	119.0 14.4	(20.3) (3.0)	187 189	109.8 14.3	(19.1) (9.5)
Speed-agility4x10 shuttle run (s)	419	14.8	(2.3)	232	13.7	(1.4)	187	14.2	(1.3)
Frequency of active travel (nº/week) *	469	4	(0,9)	251	4	(0,9)	218	4 (0,9)	
Mode of commuting º/week) * Walk Bike Car Motorcycle Bus Others	469	4 (0,9) 0 (0,0) 4 (0,9) 0 (0,0) 0 (0,0) 0 (0,0)		251	3(0,9) 0(0,0) 4(0,8) 0(0,0) 0(0,0) 0(0,0)		218	4 (0,9) 0 (0,0) 5 (0,9) 0 (0,0) 0 (0,0) 0 (0,0)	
Distance to school (m) *	324	600 (412,1000)		163	650 (450,1000)		161	600 (400,1000)	

Data are shown as mean and standard deviation, unless otherwise stated; BMI, Body mass index WC, Waist circumference; * Number of active travels to and from school per week (range: 0–10) and distance (m) expressed as Median (25th, 75th) percentile.

**Figure 1:**
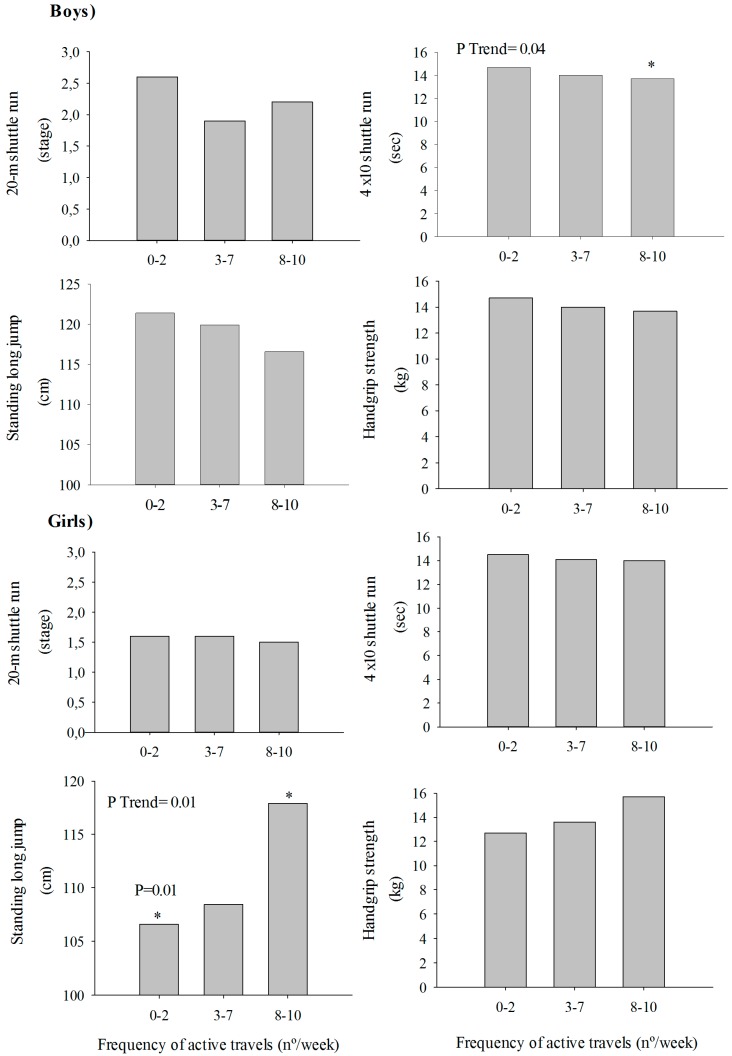
Association between active commuting to school and fitness tests, adjusting forage, BMI, and distance, from home to school for boys and girls.

The associations between active commuting to school (mainly walking) and health-related physical fitness are shown in Table 2. Active commuting to school was associated with higher scores in the speed-agility test for boys (ß = 0.062, 95% CI: 0.001 to 0.122, *p* *=* 0.048) and with higher scores in the standing long jump test for girls (ß = 1.009, 95% CI: 0.125 to 1.866, *p* *=* 0.016). The association between active commuting (three categories) to school and fitness tests is graphically shown in Figure 1. Active commuting to school was associated with higher levels in the 4 × 10 shuttle run test only in boys (*p* *=* 0.04) and in the standing long jump only in girls (*p* *=* 0.01). 

**Table 2:** Regression analysis (value of the unstandardized beta and standard error (SE)) for active commuting to school (expressed in total number of active travels to and from school per week) and fitness tests, adjusting for age, BMI, and distance.

Fitness tests *	Active Commuting						
	Boys				Girls		
	ß	SE	*p*		ß	SE	*p*
***Cardiorespiratory fitness*** 20-m shuttle run (stage)	−0.024	0.036	0.488		−0.005	0.018	0.786
***Muscular fitness*** Standing long jump (cm)	−0.495	0.297	0.435		1.009	0.433	**0.016**
Handgrip strength (kg)	−0.117	0.068	0.121		0.331	0.208	0.168
***Speed-agility*** 4 × 10 shuttle run (s)	0.062	0.031	**0.048**		−0.046	0.031	0.127

* Log-transformed data were used in the analysis for every fitness test, but raw data are shown in the table, for ß (beta) and SE (standard error).

There was an intergroup association (*p* *=* 0.01) when comparing more active commuters (8–10 active travels/week) with less active commuters (0–2 active travels/week) for the standing long jump in girls. The results remained consistent when using other categorical variables of active commuting using different cut-off points (data not shown). For the overall previous analyses, the results remained consistent when adjusting for sexual maturation status instead of age and when including sexual maturation status together with age, BMI, and distance. The results remained consistent when performing the analysis without including BMI. 

## 4. Discussion 

The main finding of this study was that boys and girls who were more active school commuters (mainly walking) had a greater speed-agility (although a slight difference) and muscle strength of the lower body, respectively, than those boys and girls who were less active school commuters. There were no significant associations between active commuting to school and other fitness variables such as cardiorespiratory fitness and upper body muscular fitness. 

### 4.1. Cardiovascular Fitness

The results from the current study suggested that there were no associations between active commuting to school and cardiorespiratory fitness levels in children.

Several studies have reported a positive association between active commuting to school and cardiorespiratory fitness levels in young people [7,11,12,13,14,15,23,24]. However, only three of these studies reported this positive association specifically for children [7,14,15]. In Danish children aged 9.7 ± 0.5 years old, cycling to school was significantly associated with higher cardiorespiratory fitness in children of both sexes. Moreover, cyclists were significantly fitter than those who walked to school or used passive transportation [7,14], possibly due to the fact that they performed a greater average trip distance to and from school than walkers. The same result was found among Norwegian children aged 9 years old [15]. It must be highlighted that in the two cross-sectional studies, the percentage of cyclists in the samples was 38.3% and 5.5%, respectively, because they belong to countries with a long previous cycling tradition. In the current study, only 0.1% of the participants cycled to school, which is not an large enough sample size to study its effects. 

On the other hand, to the best of our knowledge, there is no evidence for the association between walking to school and cardiorespiratory fitness among children. Even the three previous studies [7,14,15] did not report associations between walkers and those who used passive transportation for cardiorespiratory fitness, since there is evidence that walking as a mode of commuting seems to not be enough stimulation to modify health-related fitness [23]. Future studies should address this issue. 

The current study assessed cardiorespiratory fitness using the 20-m shuttle run test. The test uses running, which is an exercise modality to which all children and adolescents are accustomed. The test is a valid [19,24,25,26] and easily administered field test that provokes maximal effort in schoolchildren [27]. It has been used extensively to assess the aerobic fitness of children and adolescents [28]. The majority of studies that we reviewed assessed cardiorespiratory fitness using the cycle ergometry test, while we used the 20-m shuttle run test. This may be taken into account when comparing different studies.

### 4.2. Muscular Fitness

Girls who were more active commuters had higher levels of lower body muscular fitness than those who were less active commuters. There were no associations between active commuting to school and upper body muscular fitness in boys and girls.

There is little evidence on the association between active commuting to school and lower body muscular fitness in children and adolescents [9,13,15]. There were no associations between active commuting to school and muscular fitness in Danish adolescents aged 15 to 19 years old [13]. In the Danish study, the functional strength of the leg extensors was measured by the Sargent jump test, and the functional strength in the dominant arm was measured by the iron ball throw test. Moreover, there were no differences in the results between girls and boys. In Norwegian children and adolescents aged 9 to 15 years old, there was an association between active commuting to school and muscular fitness [15]. In the Norwegian study, the lower body muscular fitness was assessed by the standing long jump test, the same test used in the current study, considered a general index of both lower body and upper body muscular fitness in children and adolescents [29]. The statistical analyses included both children and adolescents and they found a sex *vs.* transport interaction. The results showed a significant association among walkers and passive females (*p* < 0.001), similar to the results in the current study. In the standing long jump test, Norwegian walkers and passive girls scored at 117 cm and 115 cm, respectively. In the current study, Spanish girls who were more active (8–10 active travels/week) scored 118 cm and had greater lower body muscular fitness compared to the less active (3–7 and 0–2 active travels/week) that scored 109 cm and 106 cm, respectively. Results from the standing fitness test showed a similar tendency in both studies, although the difference in cm was much higher in the current study. More research is needed to explain why this association was only found in girls and not in boys. 

### 4.3. Speed-Agility

Boys who were more active commuters had greater speed-agility scores than those who were less active commuters. To the best of our knowledge, there is little evidence on the association between active commuting to school and speed-agility. There were no associations between active commuting to school and speed-agility among Danish adolescents aged 15 to 19 years old [13]. In this study, the speed-agility was assessed with the 4 × 10 shuttle run test, the same test used in the current study. In the 4 × 10 shuttle run test, Danish passive and active boys scored at 10.8 s and 10.6 s, respectively. In the current study, Spanish boys who were more active commuters (8–10 active travels/week) scored 14.0 s and had greater speed-agility compared to the less active commuters (3–7 and 0–2 active travels/week) who scored 14.2 s and 14.3 s, respectively.

The positive association between active commuting and speed-agility was only reported in boys and not in girls. Since there is not a clear explanation for it, we might speculate that boys are often more competitive than girls at this age. However, more research is necessary to explain why this association was only found in boys and not in girls*.* This test is a marker of speed and agility and has been associated with bone mass in young people [1]. Extra gains in bone mass during growth could be crucial for achieving a high peak bone mass and for preventing osteoporotic fractures later in life [1].

The present observational study has some limitations, such as the study design. The questionnaire used to assess the mode and frequency of commuting to school has not been validated. However, the validity and reliability of the questionnaire is being studied. There were no data of participation in physical activity and/or sports; however, all students performed two weekly hours of physical education. Additionally, there was no information on participants’ socio-economic status (SES). Further, the current cross-sectional design does not allow us to explain the direction of the relationship between physical fitness and the mode of commuting to school. The study was conducted in January and this may underestimate the active commuting to school due to weather conditions (cold and/or rain). On the other hand, the method used to estimate home-to-school distance (*i.e.*, Google Maps) may not represent the actual route taken, as well as the short distances in home-to-school trips, but it could be recommended in order to minimize cost. Unfortunately, we were not able to measure total daily physical activity or feeding habits, which may have influenced our results. 

A major strength of the study is the use of several physical fitness tests for measuring different components of fitness and the targeting of the association between active commuting and physical fitness among schoolchildren. 

## 5. Conclusions

The present study showed that boys and girls who were more active commuters had greater speed-agility and lower body muscular fitness, respectively, than those boys and girls who were less active commuters. More studies are needed to further elucidate the complex relationships between active commuting to school and health-related physical fitness. Finally, educational campaigns to encourage active commuting should be focused on both parents and children.
